# *Kelussia odoratissima* Mozaffarian and dyslipidemia

**Published:** 2015-09-29

**Authors:** Fatemeh Dehghan Shahreza

**Affiliations:** Department of Clinical Science, School of Veterinary Medicine, Ferdowsi University of Mashhad, Mashhad, Iran

**Keywords:** *Kelussia odoratissima* Mozaff, Dyslipidemia, Bioactive components

**Implication for health policy/practice/research/medical education:**

Dyslipidemia is a major adjustable risk factor for the cardiovascular disorders and atherosclerosis progression. It is available chemoprotective ingredients that are able to provide suitable treatment with less adverse effects for dyslipidemia related complications. Kelussia odoratissima Mozaffarian of Umbelliferae family is a herbal medicine with lowering lipid effect.

## Introduction

In the last few years, attentions have been intensively directed toward natural compounds for using in chronic metabolic disorders treatment such as dyslipidemia and diabetes mellitus. There are comprehensive results of experimental and epidemiological investigations that have indicated chemoprotective effects of plant based medicines on chronic diseases. Vegetables and fruits are sources of bioactive components that have antioxidant, antidiabetic, anti-inflammatory, anticancer properties and as well as favorable impacts on health protection (1).

Dyslipidemia is a major adjustable risk factor for the cardiovascular disorders and atherosclerosis progression. In older people, lipid abnormality, diabetes, obesity and hypertension assemble to elevate the risk of micro-vascular involvement in various organs. The correction of life style by regular exercise and healthy food and suitable medicinal modalities are known to decrease the possibility of developing complications associated with lipid abnormality (2). Numerous medications are available for dyslipidemia treatment; however, none of these medicines are completely safe and without side effects. Accordingly earlier statements, it is available chemoprotective ingredients that are able to prevent dyslipidemia related complications with less adverse effects. Among various medicinal plants with anti-hyperlipidemic property, Iranian researchers have recently considered anti-inflammatory and anti-hyperlipidemia efficacy of *Kelussia odoratissima* Mozaffarian that is the newest genera of Umbelliferae family with only one species. This Iranian endangered plant found in Zagros mountain chain region.

*Kelussia odoratissima* Mozaffarian is used as a flavor vegetable in yogurt and pickle and as a herbal medicine for relieving pain, healing ulcers and amelioration of inflammation, bacterial infection, hypertension dyslipidemia and cardiovascular diseases due to contain wide range of bioactive constituents. It has been determined the essential oil of Kelussia fruit is richness of phthalides, germacrene-B, β carotenes, thirteen monoterpene hydrocarbons, sesquiterpene, steroids, Z-ligustilide, ferulic acid and germacrene-D and very unknown components that are responsible for its biological actions. *Kelussia odoratissima* aerial parts also contain high amount of piperitone epoxide and ligustilide (3). Z-ligustilide of anti-inflammatory activity is related to down- regulate the NF-κB and MAPK signal pathways in dose dependent manner; therefore, it can attenuate reactive oxygenated species generation and extracellular matrix deposition by preventing over expression of proinflammatory cytokines such as TNFα, growth factors and IL1β and inducible nitric oxide enzyme (4). Likewise, its phytostreols components are beneficial therapeutic application for lowering cholesterol level and LDL-C in individuals with and without hypercholesterolemia (5). In addition, ferulic acid is other potent constituent of Kelussia which is able to reduce collagen deposition and enhance cells survival. Its antioxidant, anti-inflammatory and antithrombotic properties, make it as a useful medicine in cardiovascular disorders (6). In the study of Shahrani et al, on Balb/c mice, was investigated *Kelussia odoratissima* Mozaffarian impact on blood fasting glucose and lipid profile. They reported, in the mice received high cholesterol diet with Kelussia 20 % hydro-ethanol extract, the serum level of cholesterol, HDL and LDL decrease significantly in compared with groups which received normal diet, high cholesterol diet and high cholesterol diet plus Kelussia 10% hydro-ethanol extract (7).

Similarly, in previous study conducted to examine lowering effect of *Kelussia odoratissima* Mozaffarian on lipid and blood fasting glucose of hyperlipidemia patients*.* These individuals were divided in control and research groups. 40 mg/day of lovastatin or 2 g/day Kelussia powder plus 40 mg/day of lovastatin were administered to control and research groups, respectively. After 14 and 30 days of the study beginning, all subjects were tested for fasting glucose, LDL-C, HDL-C and VLDL-C and results compared with data which had been provided before investigation initiation. The results indicated Kelussia consumption in hyperlipidemic patients make no significant alteration in fasting blood glucose level and lipid profile in compared with control group (8).

Although, mentioned data showed *Kelussia odoratissima* Mozaffarian is richness of bioactive components which have biological effectiveness; however, much scientific study needs to confirm anti-hyperlipidemia property of Kelussia in hyperlipidemia patients.

## Authors’ contribution

FDS is the single author of the paper.

## Conflicts of interest

The author declared no competing interests.

## Ethical considerations

Ethical issues (including plagiarism, data fabrication, double publication) have been completely observed by the author.

## Funding/Support

None.
